# Collision tumor of poorly differentiated thyroid carcinoma and intrathyroid thymic carcinoma arising in association with a branchial cleft-like cyst: a case report

**DOI:** 10.3389/fonc.2026.1900434

**Published:** 2026-08-12

**Authors:** Sayed Ali Almahari, Jehad Hammad, Khalid Khan, Haneen Alboosta, Worood Husain, Jawaher Alansari, Yusuf Barhoom

**Affiliations:** 1 Pathology Department, Salmaniya Medical Complex, Government Hospitals, Manama, Bahrain; 2 Ear, Nose and Larynx Department, Salmaniya Medical Complex, Government Hospitals, Manama, Bahrain; 3 Radiology Department, Salmaniya Medical Complex, Government Hospitals, Manama, Bahrain; 4 Surgical Department, Salmaniya Medical Complex, Government Hospitals, Manama, Bahrain; 5 General Surgery, Hepatobiliary and Transplantation Surgery, King Abdullah Medical City, Manama, Bahrain; 6 Lecturer, Arabian Gulf University, Manama, Bahrain

**Keywords:** biphasic thyroid malignancy, branchial cleft cyst, intrathyroid thymic carcinoma (ITTC), poorly differentiated thyroid carcinoma, primary squamous cell carcinoma of the thyroid

## Abstract

**Background:**

Primary squamous carcinoma of the thyroid is an exceptionally rare malignancy, and its coexistence with poorly differentiated thyroid carcinoma (PDTC) is exceedingly uncommon. The pathogenesis of thyroid squamous carcinomas remains controversial, particularly when associated with congenital epithelial remnants such as branchial cleft-like cysts or thymic/ultimobranchial remnants. We report an unusual collision tumor composed of PDTC and intrathyroid thymic carcinoma (ITTC) arising in association with a longstanding cystic thyroid lesion.

**Case presentation:**

A 59-year-old man initially presented with a left thyroid lesion that was interpreted as a benign branchial cleft cyst on ultrasound-guided fine-needle aspiration. After being lost to follow-up for more than four years, he re-presented with progressive enlargement of the lesion. Repeat cytology and core biopsy demonstrated a poorly differentiated carcinoma with squamous differentiation. Cross-sectional imaging revealed local invasion, retrosternal extension, and multiple bilateral pulmonary nodules suspicious for metastatic disease. Near total thyroidectomy was performed for symptomatic airway compression. Histopathological examination demonstrated two morphologically and immunophenotypically distinct malignant components: PDTC with solid, trabecular, and insular architecture, necrosis, increased mitotic activity, angioinvasion, lymphatic invasion, perineural invasion, and extrathyroidal extension; and a squamous carcinoma arising in association with a benign squamous-lined cyst. Immunohistochemically, the PDTC component expressed thyroglobulin, TTF-1, and PAX8, whereas the squamous component was positive for CK5/6, p63, and CD117 and negative for thyroid follicular markers, supporting ITTC.

**Outcome:**

The final diagnosis was a collision tumor composed of PDTC and ITTC arising in association with a branchial cleft-like cyst or other embryologic cystic remnant. Although pulmonary biopsy and systemic treatment were planned, the patient developed severe bronchopneumonia and died before further staging could be completed.

**Conclusion:**

This case illustrates an exceptionally rare collision tumor of the thyroid and highlights the diagnostic limitations of cytologic assessment in predominantly cystic lesions. It emphasizes the importance of repeat evaluation of enlarging cystic thyroid masses and comprehensive histopathological and immunohistochemical assessment for accurate diagnosis and appropriate clinical management.

## Introduction

Thyroid squamous carcinoma is a rare tumor, accounting for less than 1% of all thyroid malignancies (1). Even more uncommon is to find it side by side with a poorly differentiated thyroid carcinoma, particularly when associated with congenital remnants, such as branchial cleft cysts or ultimobranchial body remnants (2).

While branchial cleft cysts are typically benign congenital remnants, their potential for malignant transformation, though debated, is documented in the literature (3). Primary squamous cell carcinoma of the thyroid generally originates from one of three distinct etiologies: metaplastic transformation of a pre-existing thyroid malignancy (most commonly occurring within the context of anaplastic thyroid carcinoma), development from embryologic remnants such as the ultimobranchial body or thymic rests, or progression from pre-existing cystic lesions histologically resembling branchial cleft cysts (4).

On the other hand, poorly differentiated thyroid carcinoma (PDTC) represents an intermediate entity between well-differentiated and anaplastic thyroid carcinomas, characterized by aggressive behavior and distinct histologic criteria (5).

This case highlights a rare and diagnostically challenging scenario of a longstanding cystic lesion initially interpreted as benign, later harboring a biphasic malignancy with both PDTC and squamous carcinoma components.

## Case presentation

**Figure 1:**
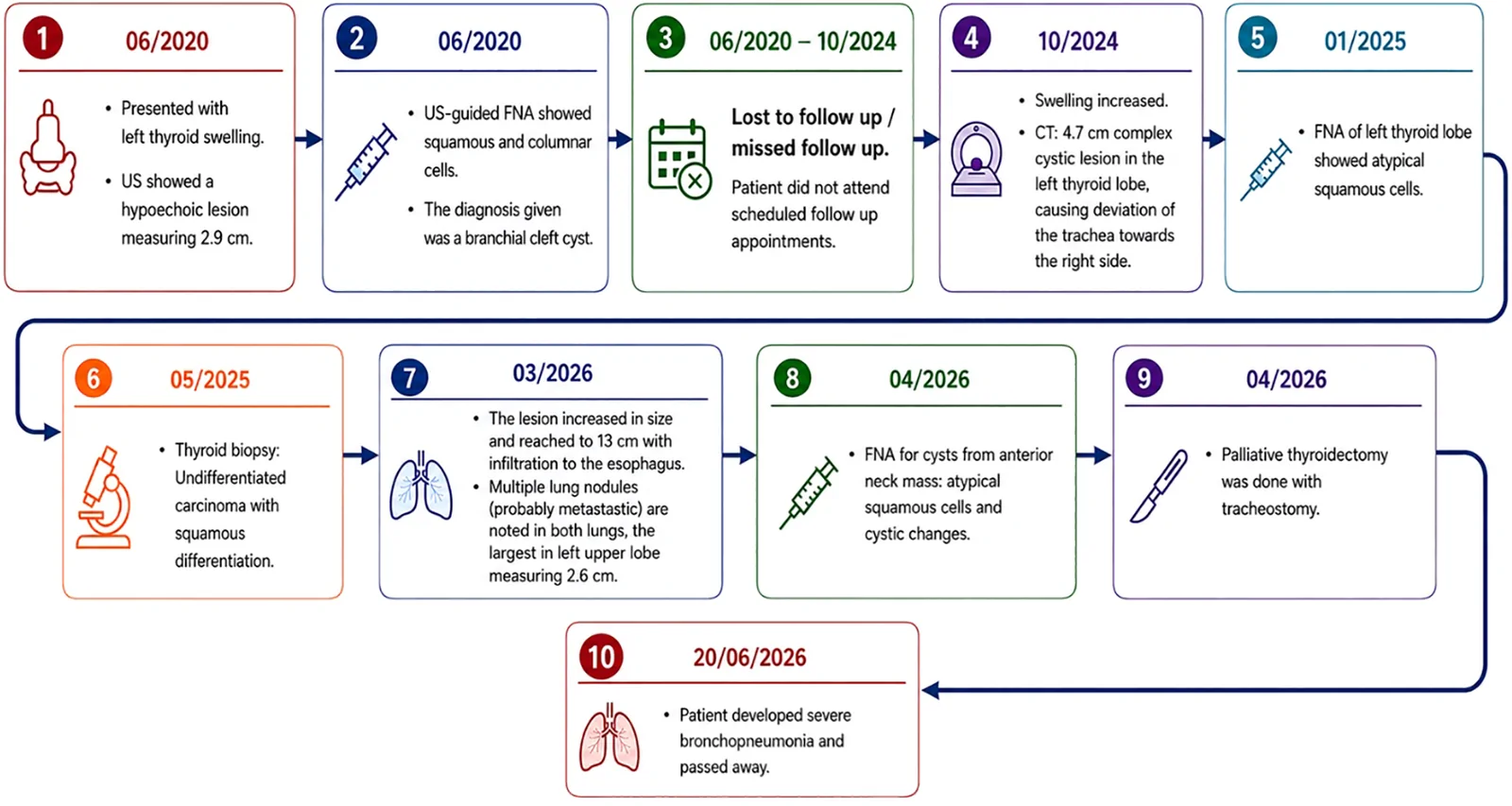
Timeline of the patient’s clinical course. Summary of the chronological sequence of clinical presentation, imaging findings, cytological and histopathological diagnoses, therapeutic interventions, and disease progression from the initial presentation in June 2020 to the patient’s death in June 2026.

**Figure 2:**
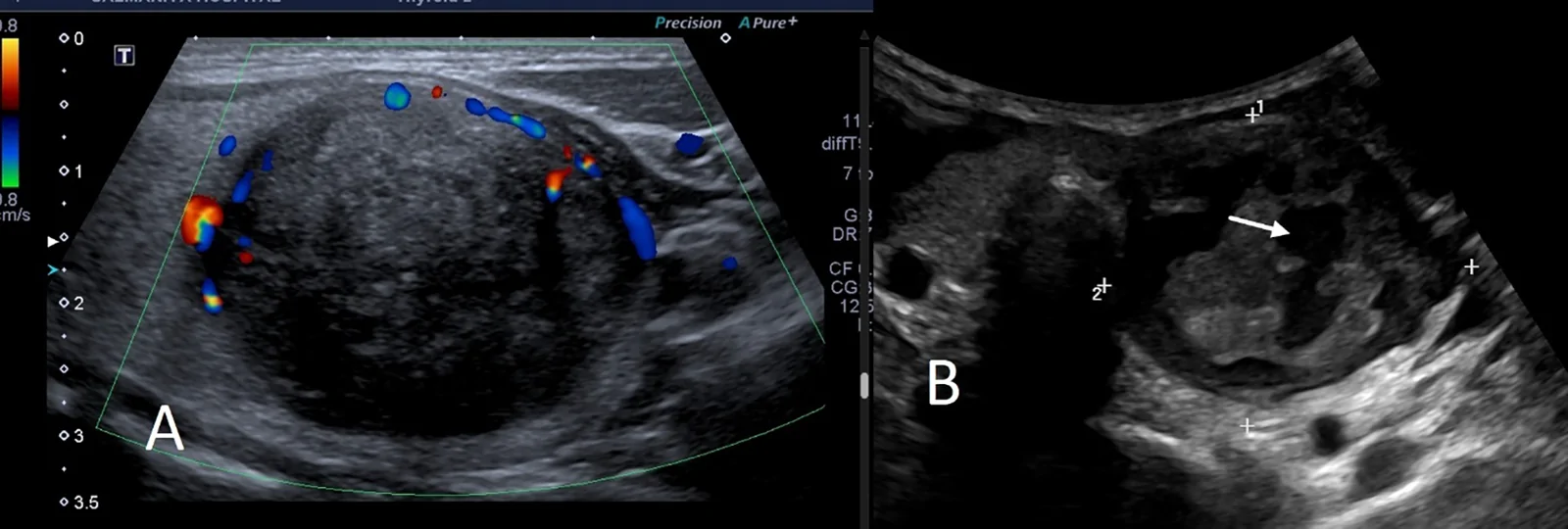
Thyroid ultrasound findings at initial presentation and follow-up. **(A)** Ultrasound examination performed at initial presentation in 2020 demonstrating a well-circumscribed hypoechoic solid lesion in the left thyroid lobe with mild peripheral vascularity on color Doppler imaging and no associated calcifications. **(B)** Follow-up ultrasound examination in 2025 showing a marked interval increase in lesion size, with a heterogeneous hypoechoic appearance and scattered cystic degenerative changes.

The patient’s clinical course is summarized in (**Figure 1**). A 59-year-old male initially presented in June 2020 with a left lower neck swelling that was clinically thought to arise from the thyroid region. Review of the ultrasound images archived in the hospital imaging system from the initial presentation demonstrated no definite cystic lesion. Instead, ultrasonography revealed a well-defined hypoechoic solid lesion within the left thyroid lobe, measuring 2.9 cm and exhibiting mild peripheral internal vascularity on color Doppler imaging and no calcifications (**Figure 2A**).

**Figure 3:**
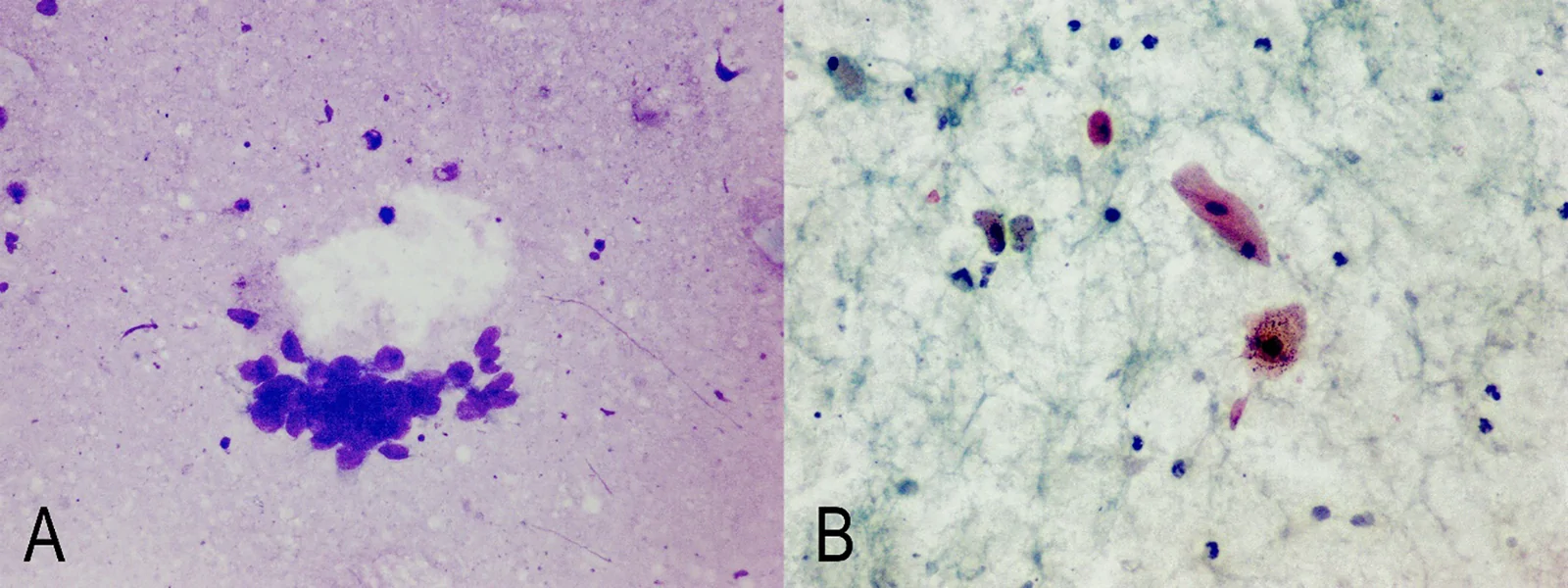
Fine-needle aspiration cytology of the thyroid cyst performed in 2020. **(A)** Direct smear demonstrating benign-appearing columnar epithelial cells without cytological atypia (May–Grünwald–Giemsa stain, ×40). **(B)** Direct smear showing squamous cells admixed with chronic inflammatory cells in the background, without significant cytological atypia (Papanicolaou stain, ×40).

Ultrasound-guided fine-needle aspiration (FNA) at that time suggested a benign cystic lesion, interpreted as a branchial cleft cyst (**Figure 3**).

**Figure 4:**
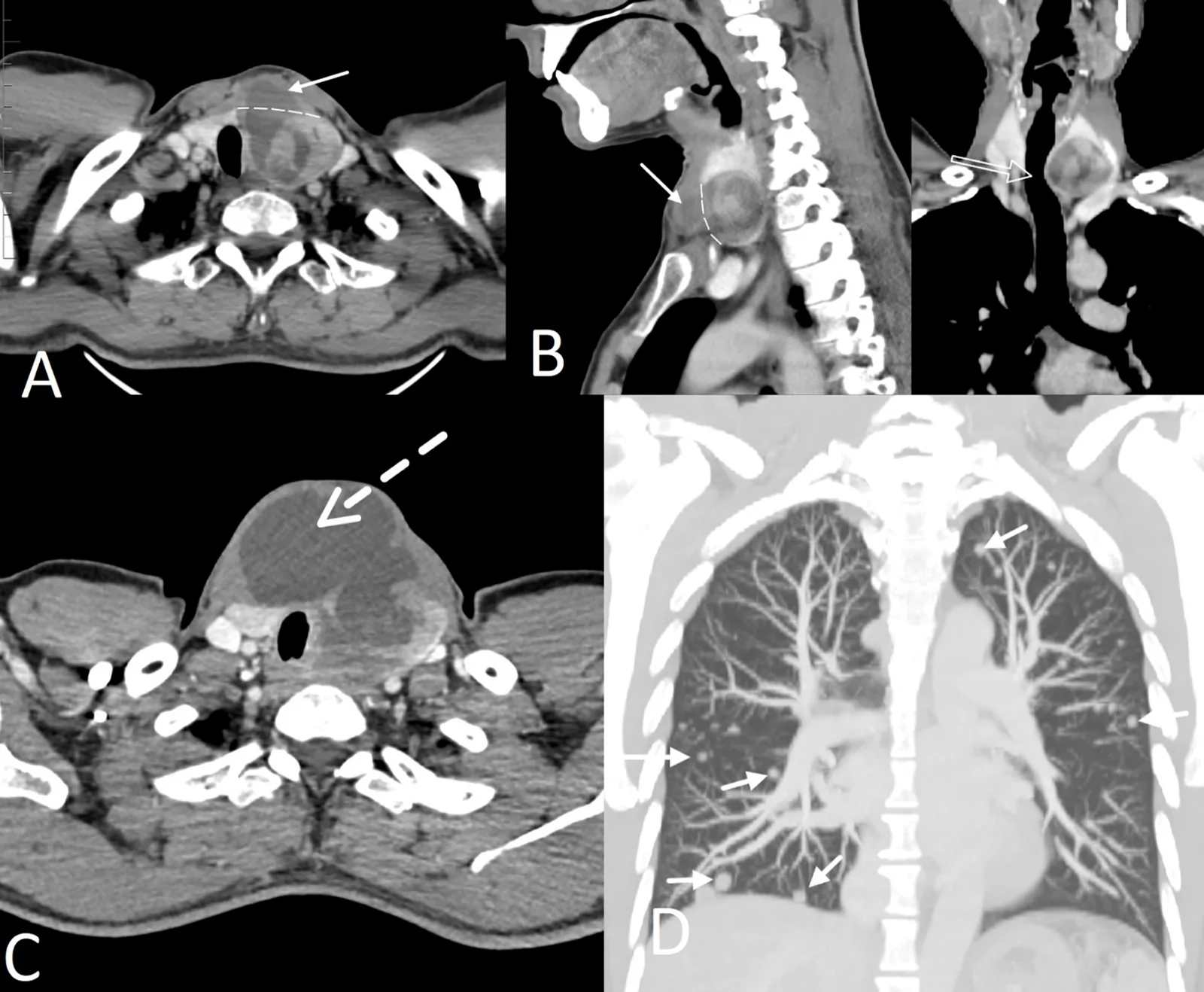
Cross-sectional imaging findings. **(A, B)** Contrast-enhanced CT of the neck in 2024 demonstrating a cystic left thyroid lesion with an enhancing solid component. The cystic component breached the anterior thyroid capsule with anteroinferior extrathyroidal extension posterior to the manubrium sterni. Mild rightward tracheal deviation and airway narrowing were present. **(C)** Follow-up contrast-enhanced CT in 2026 showing interval enlargement of the lesion, predominantly due to expansion of the inferior cystic component with retrosternal extension. The intrathyroidal and anteroinferior cystic components were contiguous and communicated with each other. **(D)** CT chest image demonstrating multiple bilateral pulmonary nodules consistent with metastatic disease. Suspected esophageal involvement on CT prompted contrast-enhanced MRI of the neck, which showed possible involvement of the left lateral esophageal wall.

Following the initial evaluation in 2020, the patient was lost to follow-up and did not re-present until late 2024. At that time, the lesion was found to have increased in size. CT imaging performed in 2024 demonstrated a cystic lesion within the left thyroid lobe containing an enhancing solid component, with a focal anterior capsular breach and extraglandular extension posterior to the manubrium sterni, associated with mild rightward tracheal displacement and airway narrowing (**Figures 4A, B**). Follow-up ultrasound in 2025 showed interval enlargement with heterogeneous hypoechoic appearance and scattered cystic degeneration (**Figure 2B**). Due to continued growth, repeat FNA and a core biopsy were performed in mid-2025, both revealing a poorly differentiated carcinoma with squamous differentiation, prompting surgical intervention. Subsequent CT imaging in 2026 demonstrated further enlargement of the lesion with marked increase in the inferior cystic component and retrosternal extension (**Figure 4C**). The cystic lesions appeared contiguous and communicating, and multiple bilateral pulmonary nodules suspicious for metastatic disease were identified. MRI of the neck further raised suspicion for involvement of the left lateral esophageal wall (**Figure 4D**).

The patient subsequently underwent near total thyroidectomy with excision of adjacent structures as palliative treatment due to compressive symptoms, including impaired breathing resulting from local mass effect.

**Figure 5:**
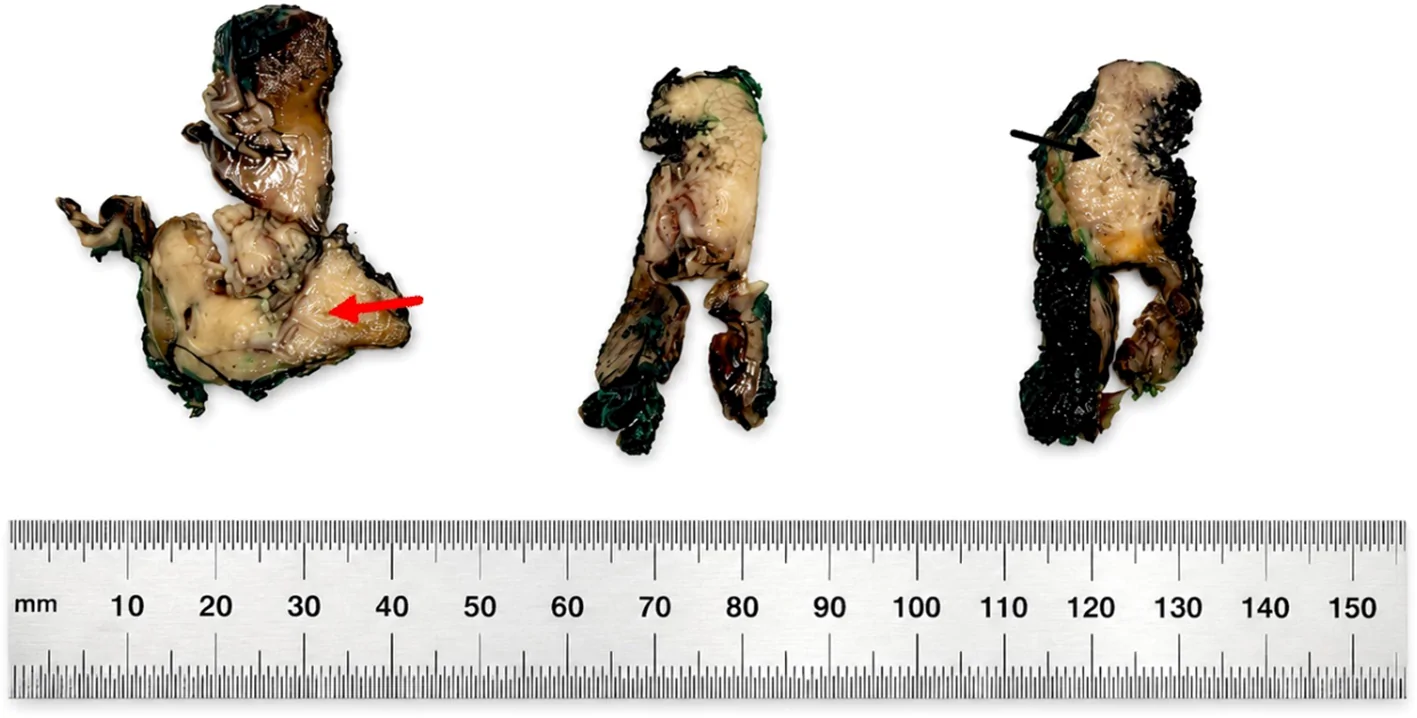
Gross examination of representative sections from the left thyroid lobe. Three representative slices are shown. The left slice demonstrates a cystic lesion with surrounding white-tan necrotic tumor, corresponding to the squamous cell carcinoma component (red arrow). The right slice shows a solid pale-white tumor area corresponding to the poorly differentiated thyroid carcinoma component (black arrow).

Gross examination demonstrated a near total thyroidectomy specimen containing two cystic lesions, including a large superior cyst and a smaller inferior cyst. A dominant infiltrative tumor measuring 7 cm in greatest dimension involved the left thyroid lobe predominantly, extending into the isthmus and right lobe, and exhibited areas of necrosis and cystic change (**Figure 5**).

**Figure 6:**
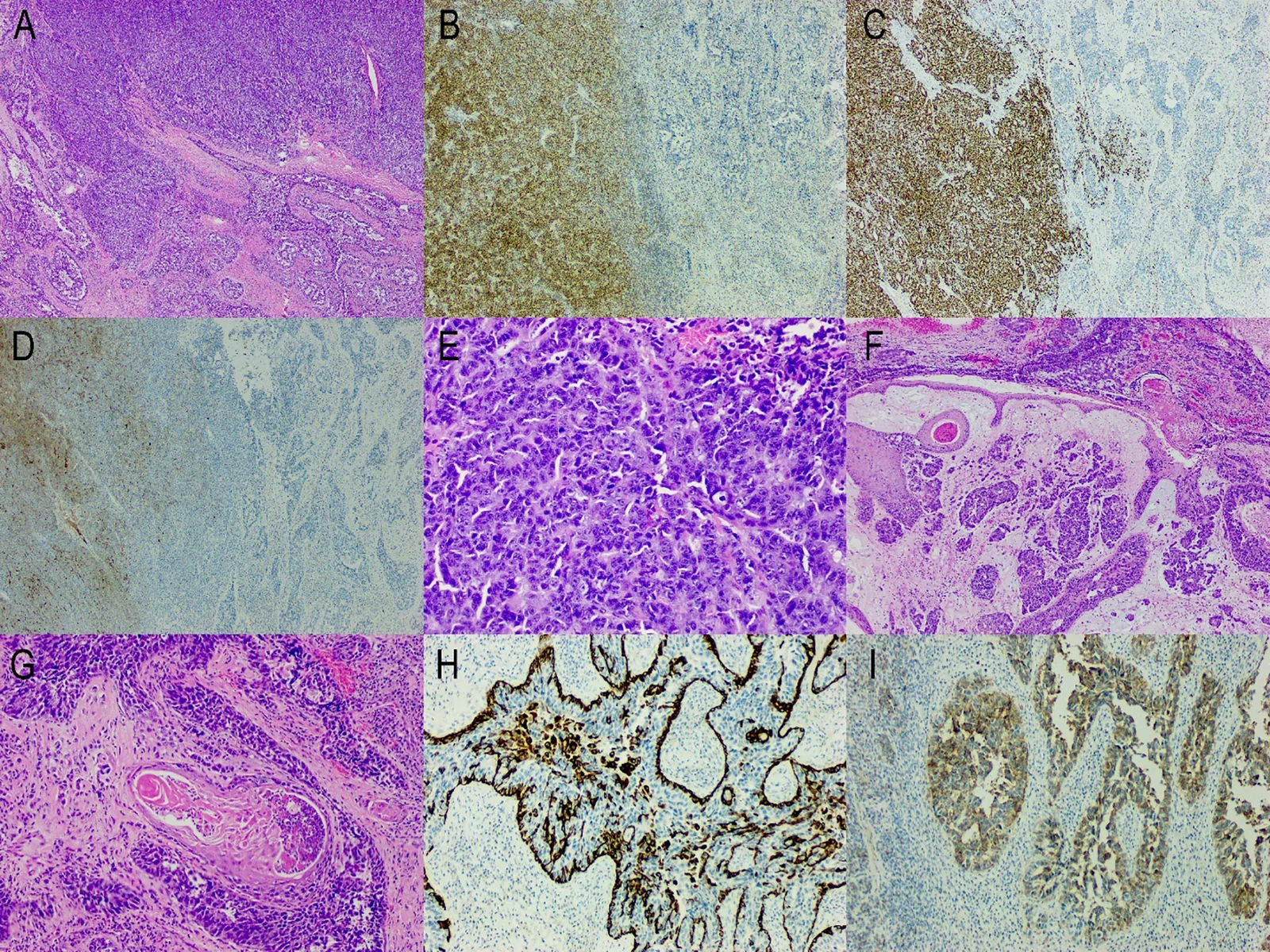
Histopathological and immunohistochemical features of the tumor. **(A–D)** Representative sections demonstrating the biphasic nature of the tumor, with the poorly differentiated thyroid carcinoma (PDTC) component on the left side of the image and the squamous cell carcinoma (SCC) component on the right side. The PDTC component is composed of solid nests, trabeculae, and sheets of monotonous tumor cells with a high nuclear-to-cytoplasmic ratio and increased mitotic activity. Immunohistochemically, the PDTC component is positive for PAX8, TTF-1 and thyroglobulin. While the squamous cell carcinoma is negative for the three markers (H&E ×2, PAX8 ×4, TTF-1 ×4, and thyroglobulin ×4). **(E)** Higher-power view of the PDTC component showing a trabecular and solid growth pattern, marked nuclear atypia, and increased mitotic activity (H&E ×20). **(F–I)** The SCC component arising in association with a benign squamous-lined cyst, showing a spectrum from squamous dysplasia to invasive squamous cell carcinoma. The SCC component demonstrates immunoreactivity for CK5/6 and CD117 (H&E ×4, H&E ×20, CK5/6 ×10, and CD117 ×10).

Histological examination revealed a biphasic malignant neoplasm. The predominant component consisted of poorly differentiated thyroid carcinoma (PDTC), characterized by solid, trabecular, and insular growth patterns, increased mitotic activity, necrosis, and immunoreactivity for thyroglobulin, TTF-1, and PAX8. The second component was sharply demarcated and composed of nests and sheets of atypical squamous cells with keratinization and intercellular bridges, showing positivity for CK5/6 and p63 while lacking expression of thyroid markers (**Figures 6A-I**). Both components were entirely negative for synaptophysin, chromogranin A, calcitonin, and BRAF V600E immunohistochemistry. TP53 shows a wild-type pattern of staining.

The squamous component appeared to arise in association with cystic structures (**Figure 6F**), raising the possibility of origin from a branchial cleft-like cyst or thymic/ultimobranchial remnant. The tumor demonstrated angioinvasion, lymphatic invasion, and perineural invasion in the PDTC component, as well as extrathyroidal extension involving the pre-tracheal fascia. The PDTC component involved the surgical margins, whereas the squamous carcinoma component remained confined (R1 excision) to the thyroid gland. The final pathological diagnosis was a biphasic malignancy composed of poorly differentiated thyroid carcinoma and ITTC/squamous cell carcinoma, staged at least (Clinical/Radiological staging was pT4a N0 M1).

Following multidisciplinary discussion at the tumor board (MDT), the consensus recommendation was to proceed with fluorodeoxyglucose positron emission tomography/computed tomography (FDG PET-CT) for comprehensive staging and assessment of disease extent. Given the presence of a pulmonary lesion, image-guided biopsy was advised to determine whether it represented metastatic poorly differentiated thyroid carcinoma (PDTC) or a squamous cell carcinoma (SCC), as identification of the metastatic component would have important therapeutic implications. Subsequent management and systemic treatment would therefore be tailored according to the histopathological and immunophenotypic findings of the biopsied lung lesion.

In addition, the MDT recommended administration of radioactive iodine (RAI) therapy, recognizing that PDTC is often poorly iodine-avid and therefore may have a limited likelihood of response; however, RAI was considered reasonable to provide any potential therapeutic benefit. The SCC component involving the neck was also planned to be treated with external beam radiotherapy. Overall management would be individualized based on the results of further staging investigations and tissue characterization of the pulmonary lesion. 

Unfortunately, the patient passed away with severe bronchopneumonia without performing the lung biopsy.

## Discussion

This case represents a rare and complex diagnostic example of a biphasic thyroid malignancy arising from a longstanding cystic lesion, initially interpreted as a branchial cleft cyst. The clinical course of this case reflects a recognized diagnostic pitfall in head and neck pathology, whereby cystic lesions, particularly those enlarging over time, may harbor or evolve into malignancy. The initial benign cytological interpretation in 2020 likely reflects either sampling limitations inherent to cystic lesions or the possibility that malignant transformation had not yet occurred.

To our knowledge, no previous case describing the coexistence of poorly differentiated thyroid carcinoma (PDTC) and intrathyroid thymic carcinoma (ITTC) arising in association with a branchial cleft-like cyst or similar embryologic cystic remnant has been reported in the English-language literature. Consequently, the pathogenesis can only be inferred from the known biology of each individual tumor and their relationship to embryologic remnants rather than direct comparison with previously published cases.

The presence of a distinct squamous cell carcinoma component within the thyroid raises important considerations regarding tumor origin. Primary squamous cell carcinoma of the thyroid is extremely rare, and exclusion of metastatic disease or direct extension from adjacent organs is mandatory (6). In this case, the intimate association of the squamous cell carcinoma with positivity for p63 and CK5/6, together with the presence of cystic spaces lined by benign stratified squamous epithelium, supports origin from a non-follicular epithelial source, such as a branchial cleft–like cyst or a thymic/ultimobranchial remnant. Strong CD117 positivity further supports thymic differentiation, consistent with ITTC, even in the absence of CD5 expression, which is known to be variably expressed in such tumors (7).

Equally significant is the presence of PDTC, a follicular-derived malignancy with aggressive biological behavior (5). The two tumor components in this case are morphologically and immunophenotypically distinct, with a clear demarcation between them. This finding favors a collision tumor or dual-origin process rather than divergent differentiation within a single neoplasm. The PDTC component demonstrates numerous high-risk features, including high mitotic activity, necrosis, angioinvasion, lymphatic invasion, perineural invasion, and extrathyroidal extension, all of which are associated with poor prognosis (8).

The distinction between a collision tumor and a composite tumor deserves clarification. Collision tumors represent the coexistence of two histogenetically distinct neoplasms that arise independently and subsequently become juxtaposed, whereas composite tumors arise from a common neoplastic precursor and exhibit divergent differentiation. In the present case, the two components demonstrated distinct morphologic features, mutually exclusive immunophenotypes, and a sharp interface without transitional areas. Furthermore, the squamous carcinoma appeared to arise in association with a benign squamous-lined cyst, whereas the PDTC showed conventional follicular epithelial differentiation. These findings favor a collision tumor rather than a composite neoplasm, although definitive proof of independent clonal origin would require molecular analysis.

The longstanding cystic lesion may provide an important clue to the pathogenesis of this unusual neoplasm. While the precise nature of the cyst cannot be established with certainty, the presence of benign squamous epithelium contiguous with the ITTC component raises the possibility of origin from a branchial cleft-like cyst or another embryologic remnant such as thymic or ultimobranchial tissue. Alternatively, the cystic lesion may simply have represented the earliest manifestation of the squamous component before overt malignant transformation became apparent. Regardless of the exact origin, the prolonged interval between the initial benign cytological diagnosis and subsequent development of an aggressive malignancy underscores the limitations of cytological assessment in predominantly cystic lesions.

Initially, the possibility of anaplastic thyroid carcinoma with squamous differentiation was considered because PDTC may progress to anaplastic carcinoma during tumor evolution. However, several findings argue against this interpretation. The tumor demonstrated a prolonged clinical history spanning several years rather than the rapid progression typical of anaplastic thyroid carcinoma. Furthermore, the squamous component remained morphologically distinct from the PDTC, exhibited a separate immunophenotype, and retained a wild-type TP53 immunohistochemical staining pattern, making dedifferentiation from PDTC less likely (9).

From a clinical perspective, this case emphasizes the need for continued surveillance of adult cystic neck lesions that demonstrate progressive enlargement or atypical radiological features despite apparently benign cytology. Repeat imaging, repeat fine-needle aspiration or core biopsy, and consideration of surgical excision should be undertaken whenever clinical suspicion persists. Extensive histological sampling and comprehensive immunohistochemical characterization remain essential for identifying multiple coexisting tumor components that may otherwise be overlooked.

Management of this patient was particularly challenging because of positive surgical margins, extensive local invasion, and radiologically suspected pulmonary metastases. Radioactive iodine therapy was considered because of the follicular-derived PDTC component, although its effectiveness was expected to be limited given the relatively poor iodine avidity of PDTC (10). Since immunohistochemistry demonstrated absence of BRAF V600E expression, treatment with BRAF-targeted therapy was not indicated (11). Comprehensive molecular profiling remains advisable in metastatic PDTC to identify potentially targetable alterations, including RET and NTRK fusions, which may permit treatment with selective kinase inhibitors (11, 12). In the absence of actionable molecular alterations, lenvatinib remains the preferred first-line systemic therapy for progressive radioiodine-refractory disease, with sorafenib representing an alternative option (10–12). External beam radiotherapy would also be appropriate to improve locoregional control in view of the positive margins and extrathyroidal extension (13). Importantly, confirmation of the metastatic component by biopsy would have significant therapeutic implications, as metastatic ITTC may require treatment strategies different from those employed for metastatic PDTC.

Overall, this case illustrates the remarkable biological heterogeneity that may occur within thyroid malignancies and highlights the importance of integrating clinical history, imaging findings, morphology, and immunophenotypic features to achieve an accurate diagnosis. It also emphasizes the need to distinguish true collision tumors from composite neoplasms, as this distinction provides important insights into tumor pathogenesis and may ultimately influence diagnostic interpretation and future molecular investigations.

## Conclusion

This case describes an exceptionally rare biphasic thyroid malignancy composed of poorly differentiated thyroid carcinoma and a squamous carcinoma with features of ITTC, arising in association with a longstanding cystic lesion initially interpreted as a branchial cleft cyst. The coexistence of two morphologically and immunophenotypically distinct malignant components supports a complex pathogenesis involving embryologic remnants or malignant transformation within a pre-existing cystic lesion. The case highlights the diagnostic limitations of cytologic sampling in predominantly cystic lesions and emphasizes the need for repeat assessment of enlarging or clinically atypical cystic neck masses. Accurate diagnosis requires meticulous histologic evaluation, extensive sampling, and judicious use of immunohistochemistry. Recognition of these rare collision tumors is essential because of their distinct biological behavior, prognostic implications, and therapeutic considerations, particularly when metastatic disease is present.

## Data Availability

The original contributions presented in the study are included in the article/supplementary material. Further inquiries can be directed to the corresponding author.
